# Protocol: optimisation of a grafting protocol for oilseed rape (*Brassica napus*) for studying long-distance signalling

**DOI:** 10.1186/s13007-016-0122-x

**Published:** 2016-03-25

**Authors:** Anna Ostendorp, Steffen Pahlow, Jennifer Deke, Melanie Thieß, Julia Kehr

**Affiliations:** Molecular Plant Genetics, University Hamburg, Biocenter Klein Flottbek, Ohnhorststr. 18, 22609 Hamburg, Germany

**Keywords:** *Brassica napus*, Grafting, Micrografting, Rootstock, Scion, Hydroponic culture

## Abstract

**Background:**

Grafting is a well-established technique for studying long-distance transport and signalling processes in higher plants. While oilseed rape has been the subject of comprehensive analyses of xylem and phloem sap to identify macromolecules potentially involved in long-distance information transfer, there is currently no standardised grafting method for this species published.

**Results:**

We developed a straightforward collar-free grafting protocol for *Brassica napus* plants with high reproducibility and success rates. Micrografting of seedlings was done on filter paper. Grafting success on different types of regeneration media was measured short-term after grafting and as the long-term survival rate (>14 days) of grafts after the transfer to hydroponic culture or soil.

**Conclusions:**

We compared different methods for grafting *B. napus* seedlings. Grafting on filter paper with removed cotyledons, a truncated hypocotyl and the addition of low levels of sucrose under long day conditions allowed the highest grafting success. A subsequent long-term hydroponic cultivation of merged grafts showed highest survival rates and best reproducibility.

**Electronic supplementary material:**

The online version of this article (doi:10.1186/s13007-016-0122-x) contains supplementary material, which is available to authorized users.

## Background

Grafting is a well-established technique for joining vegetative tissues from two or more plants. It has been widely applied to improve the properties of different vegetables and other horticultural crop plants [1]. Key for a successful establishment of graft unions is the formation of a continuous vascular system between the grafting partners that are usually called scion (shoot part of the graft) and rootstock (root part of the graft). Therefore, grafting is most successful in dicotyledonous plants possessing a vascular cambium and more difficult or even impossible in monocotyledonous plants.

Because of the reunion of functional xylem and phloem connections, grafting has also become an excellent experimental tool to study long-distance mobility of a wide range of molecules in living plants [2]. Grafting studies have provided conclusive evidence that long-distance transport is involved in diverse, but likewise important, physiological processes. Examples are the photoperiodic regulation of flowering [3, 4], the systemic spread of viruses [5, 6], phytohormone transport and action [7], apical dominance [8], nodule formation [9], small RNA movement [10–12], systemic acquired resistance [13, 14], and systemic gene silencing [15]. Grafting methods for confirming long-distance transport of regulating molecules are established for a wide range of plant species, including *Nicotiana benthamiana* [16, 17], *Medicago truncatula* [18], and the model species *Arabidopsis thaliana* [19–24]. These techniques have been applied successfully to study signal transduction, for example by micro RNAs (miRNAs) [10, 12, 25, 26].

*Brassica napus* is a suitable plant for studying long-distance communication, because it allows obtaining phloem and xylem exudates in sufficient amounts for analysing sap compositions. In this species that is related to the model plant *A. thaliana*, sampling is relatively easy, and sample volumes are comparably large [10–12, 27–29].

Several studies identified hundreds of proteins and small RNAs (smRNAs) in phloem sap of *B. napus* [28, 30, 31]. To verify their long-distance mobility in vivo, so far grafting studies between wild-type and mutants/overexpressor plants were performed in Arabidopsis [10, 12]. The major reasons are that Arabidopsis transformation is straightforward and unmatched genetic resources are publicly available. However, the use of Arabidopsis in grafting experiments to study phloem mobility does only allow indirect conclusions about the mobility of phloem-localised molecules, since phloem sampling and, thus, direct measurements of changing compound levels in phloem sap are hardly possible. Therefore, a system allowing the same type of analysis of phloem long-distance signalling in Brassica would be desirable. Although not as easy as in Arabidopsis, several reports describe the successful transformation of *B. napus* using *Agrobacterium tumefaciens* [32, 33] what should allow the creation of suitable transgenic plants for grafting experiments. However, up to now only a few not very detailed grafting protocols for this species have been published in international journals [34–36], and no information about efficiency and reproducibility of grafting is documented.

This study attempts to provide a robust collar-free grafting procedure for different *B. napus* cultivars that is useful to confirm long-distance phloem mobility of potential signalling compounds identified in isolated phloem sap from this species. We describe an optimised flat-surface root-to-shoot grafting protocol for *B. napus* seedlings. The established grafting method does not require a collar to support the graft union and enabled a high short-term (up to 100 %) and a reasonable long-term survival rate (70–80 %) after the transfer to hydroponic culture or soil, respectively, indicating a high ability for the formation of functional vascular connections. This was confirmed by following the movement of the phloem-specific fluorescence dye carboxyfluorescein diacetate (CFDA) through the established graft unions.

## Methods

### Reagents

- Seed sterilisation solution (see REAGENT SETUP)
- Sodium hypochloride (Applichem-Panreac, cat. no. 213322.1214)
- Tween-20 (Applichem-Panreac, cat no. A1389,0500)
- 70 and 100 % ethanol (Applichem-Panreac, cat. no. A3678,1000)
- Murashige and Skoog (MS) medium (Duchefa, cat. No. M0245.0050)
- Sucrose (Applichem-Panreac, cat. no. A3935,5000)
- Agarose (BD, cat. no. 214010)
- Sterile deionised water
- Hydroponic medium (see REAGENT SETUP)
- Carboxyfluorescein diacetate (CFDA) (Sigma-Aldrich, cat. no. 21879-100MG-F)
- Dimethylsulfoxide (Applichem-Panreac, cat. no. A1584,0500)
- Einheitserde Classic (Einheitserdewerk Uetersen).

### Equipment

- Tweezers
- Petri dishes (92 × 15 mm, Sarstedt, cat. no. 82.1473)
- Growth chamber set to 22 °C, 70 % humidity, light conditions are dependent of the step in the grafting protocol (see PROCEDURE)
- binocular (SZ40, Olympus)
- round filter paper (90 mm, GE Life Science, cat. no. 1440-090)
- razor blades (Wilkinson Sword Classic)
- 50 ml conical tubes (Sarstedt, cat. no. 62.547.254)
- fluorescence stereomicroscope (MZ FL III, Leica)
- parafilm (Bemis, cat. no. #PM999)
- laminar flow tissue culture cabinet
- 4 °C standard laboratory fridge.

### Reagent setup

- **Seed sterilisation solution** 7 % (v/v) sodium hypochloride, 0.05 % (v/v) Tween-20
- **Germination plate** ½ Murashige and Skoog (MS) medium, 1 % agarose
- **Regeneration plates** (1) ½ MS medium, 0.5 % (w/v) sucrose, 1 % agar, (2) ½ MS, 1 % agar, (3) filter paper moistened with 0.5 % sucrose, (4) filter paper with sterile water
- **Hydroponic medium** the medium is described in Buhtz et al. [12]: 0.6 mM NH_4_NO_3_, 1 mM Ca(NO_3_)_2_, 0.04 mM Fe-EDTA, 0.5 mM K_2_HPO_4_, 0.5 mM K_2_SO_4_, 0.4 mM Mg(NO_3_)_2_. Micro nutrients added: 0.8 μM ZnSO_4_, 9 μM MnCl_2_, 0.1 μM Na_2_MoO_4_, 23 μM H_3_BO_3_, 0.3 μM CuSO_4_. The pH was adjusted to 4.7 with 37 % HCl
- **CFDA solution** 5 mg CFDA were solved in 100 µl 100 % DMSO. For plant application a 1:100 dilution in deionized water is used.
- ***Brassica napus*seeds** can be obtained commercially and from various breeders and research laboratories. This protocol is optimised for the Drakkar and Licosmos cultivars.

### Procedure

#### Critical step

Good sterilisation is necessary to prevent bacterial or fungal contaminations during graft recovery. All work should be done under a sterile laminar flow cabinet.

#### Seed sterilisation and germination

Timing ~1 h (for sterilisation, depending on the amount of seeds), 6 days (for germination)

1. Sterilisation solution should be prepared freshly. Seeds are incubated for 2 min in 2 ml 100 % ethanol and subsequently surface sterilised with 2 ml sterilisation solution for 15 min and then washed with sterile water three times for 10 min [37]. A better washing can be achieved by shaking the reaction tube containing the seeds.
2. Transfer the sterilised seeds to the germination plate (6–8 seeds per plate) and incubate the plates at 4 °C in the dark for 3 days in a vertical orientation.
3. After 3 days incubate the plates under short day conditions (light: 8 h, dark: 16 h, light intensity 100 μmol m^−2^ s^−1^, 22 °C and a relative humidity of 70 %) in a growth chamber and store the plates in a vertical orientation. After approx. 6 days old 3–5 cm long seedlings were used for grafting.

#### Plant grafting

Timing ~1 h (for 40 grafts), 14 days (for regeneration)

4. Cut up to four seedlings on a cutting plate (petri dish with a round Whatman filter paper moistened with sterile water) with a razor blade under a binocular microscope. Remove cotyledons as well as 1–2 cm of the middle of the hypocotyl of the seedlings (Fig. 1).

**Fig. 1:**
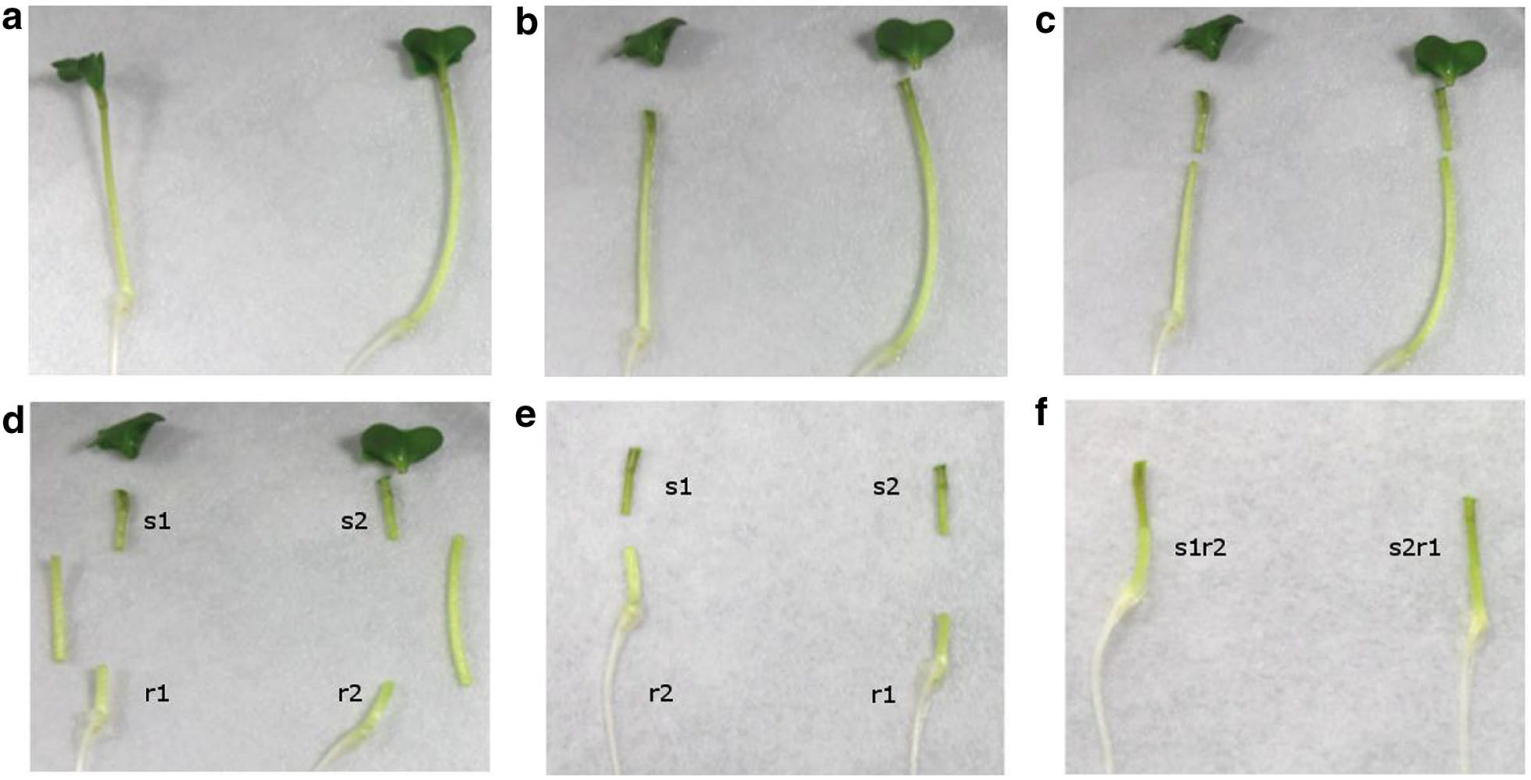
Grafting procedure of *B. napus* seedlings. 3 day old *B. napus* seedlings were first transferred to a cutting plate covered with moistened filter paper (**a**). After removing of the cotyledons with a razor blade (**b**), 1–2 cm of the middle of the hypocotyls where removed (**c**, **d**). Finally the cut plant parts [shoot (*s1* and *s2*) and root (*r1* and *r2*)] were transported to a regeneration plate (**e**) and joined using tweezers (**f**). Therefore the shoot of the one seedling (*s1*) were connected to the root of the other plant (*r2*)
5. Join the cut plant parts on a regeneration plate with the respective regeneration conditions using forceps. Close the regeneration plates with parafilm and incubate them under short day conditions with plates in a vertical orientation (5°–10°). Check the grafts after 6, 10 and 14 days.

#### Critical step

In contrast to the protocol from Marsch-Martinez [24] optimal cutting of hypocotyls and cotyledons can be achieved on a moistened filter paper, propably due to the higher stiffness of *B. napus* plants compared to *A. thaliana*. Another difference to this protocol is the removal of the central part of the hypocotyl. In Brassica, the fast longitudinal growth of the seedlings hindered a successful graft formation when this step was omitted. Attention needs to be paid when joining the cut scion and root that no water film is within the parts. Ecotypes with an increased longitudinal growth direction like Drakkar should be regenerated for 14 days since the graft junction is fairly instable. Ecotypes like Licosmos with a reduced longitudinal growth can be regenerated for only 10 days.

#### Post-grafting cultivation

Timing 14 days (for regeneration), 30 min (for CFDA monitoring)

6. For hydroponic cultivation 14 day old grafts are transferred to 50 ml conical tubes (Fig. 3a) and grown in boxes using the hydroponic medium described in Buhtz et al. [12]. For this purpose, wrap black foam around the grafted plant with the graft junction located in the middle of the foam. Cover the sides of the conical tubes with aluminium foil and fill in 40 ml of hydroponic medium to reduce algal growth.
7. Place the wrapped graft in the conical tube in such a way that the foam does not get in contact with the medium, but holds the graft in place and store the tubes in a rack (Additional file 3: Figure S3).
8. Place the rack in a standard polystyrene box with at least the same height or higher than the grafted plants. A box of 32 cm × 25 cm × 17 cm (length, width, height) is sufficient for cultivation of ca. 30 plants (Additional file 3: Figure S3). Cover the box with a light permissive plastic cover. For a simple set-up use plastic wrap and puncture up to 20 small holes for ca. 12 plants to allow adequate aeration. Cultivate the covered grafts for 10–14 days.
9. For soil cultivation the plants should be well-watered. Cover the transferred grafts with a plastic cover to avoid dehydration of the plantlets. Grafts are cultivated under long day conditions in a growth chamber (light: 16 h, dark: 8 h, light intensity of 80 μmol m^−2^ s^−1^, 22 °C and a relative humidity of 60 %; Fig. 3). After 14 days plants can be grown in the greenhouse either hydroponically in single pots or on soil.

#### Critical step

If hydroponic growth is favoured, precautions have to be taken to reduce algal growth. Therefore cover all conical tubes with aluminium foil. Also ensure that the graft junction has no contact to the hydroponic medium to minimise the formation of adventitious roots. Puncturing the cover lid or loosely covering the plants helps to achieve an appropriate aeration without letting the plantlets dry out. Chen et al. [37] describe a similar method for Arabidopsis grafts, but due to the larger size of grafted *B. napus* plants a floating system as described is not applicable. Furthermore we observed that taking care that the foam is not in contact with the hydroponic medium avoids the formation of adventitious roots. To prevent a drying-out of freshly transferred grafts to soil or hydroponic medium it is advisable to cover those plants with a light permissive plastic cover, in addition to the high humidity set in the growth chamber.

10. The success of the formation of functional vascular connections within the grafts can be monitored using carboxyfluorescein diacetate, a phloem-specific fluorescence dye, as described in Grignon et al. 1989 [38]. Grafted plants were transferred to agar plates containing ½ MS and 1 % (w/v) agar to prevent drying-out. One leave per plant is punctured and a few microliters of a 10 µM CFDA solution are applied. After an incubation of 30 min at ambient temperature, fluorescence can be observed under a fluorescence stereomicroscope equipped with a GFP filter (Fig. 4).

## Results and discussion

Grafting conditions were optimised for *B. napus* seedlings to improve the survival rate of grafts. In contrast to other studies, we followed grafting success over a longer time period until grafted plants were successfully transferred to hydroponic culture or to soil. Obviously, success rates are lower at a later time-point than after a few days. However, since our goal was to transfer stable grafts to hydroponic culture or soil to let the plants grow until sampling of phloem and xylem sap is possible, it is the more meaningful measure in this case. Plants with a non-functional vascular system are prone to die within 2 weeks of post-grafting cultivation. Since initial experiments showed that graft formation was more successful under short day than under long day conditions, all further experiments were performed in growth chambers with 8 h light and 16 h dark.

**Fig. 2:**
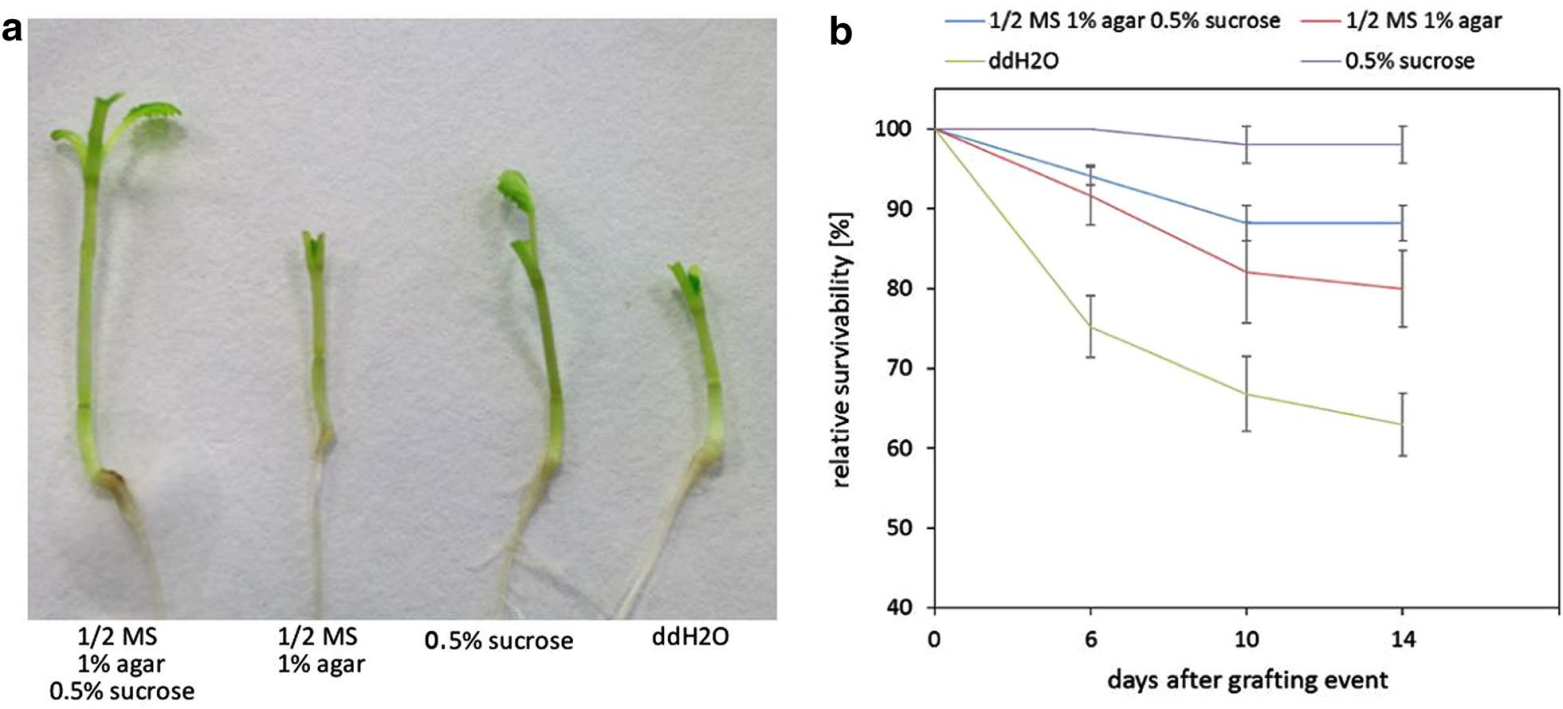
14 day old grafts of *B. napus* cv. Drakkar after different regeneration conditions. **a** Overview of successfully grafted plants regenerated under different conditions after 14 days. The *arrow* indicates the graft junction. **b** Survival rate of grafted plants upon different regeneration conditions: ½ MS 1 % agar 0.5 % sucrose (n = 53); ½ MS 1 % agar (n = 63); ddH_2_O (n = 91); 0.5 % sucrose (n = 51). Grafting success was investigated after 6, 10 and 14 days and plotted is the relative survivability of grafted plants under different regeneration conditions

**Fig. 3:**
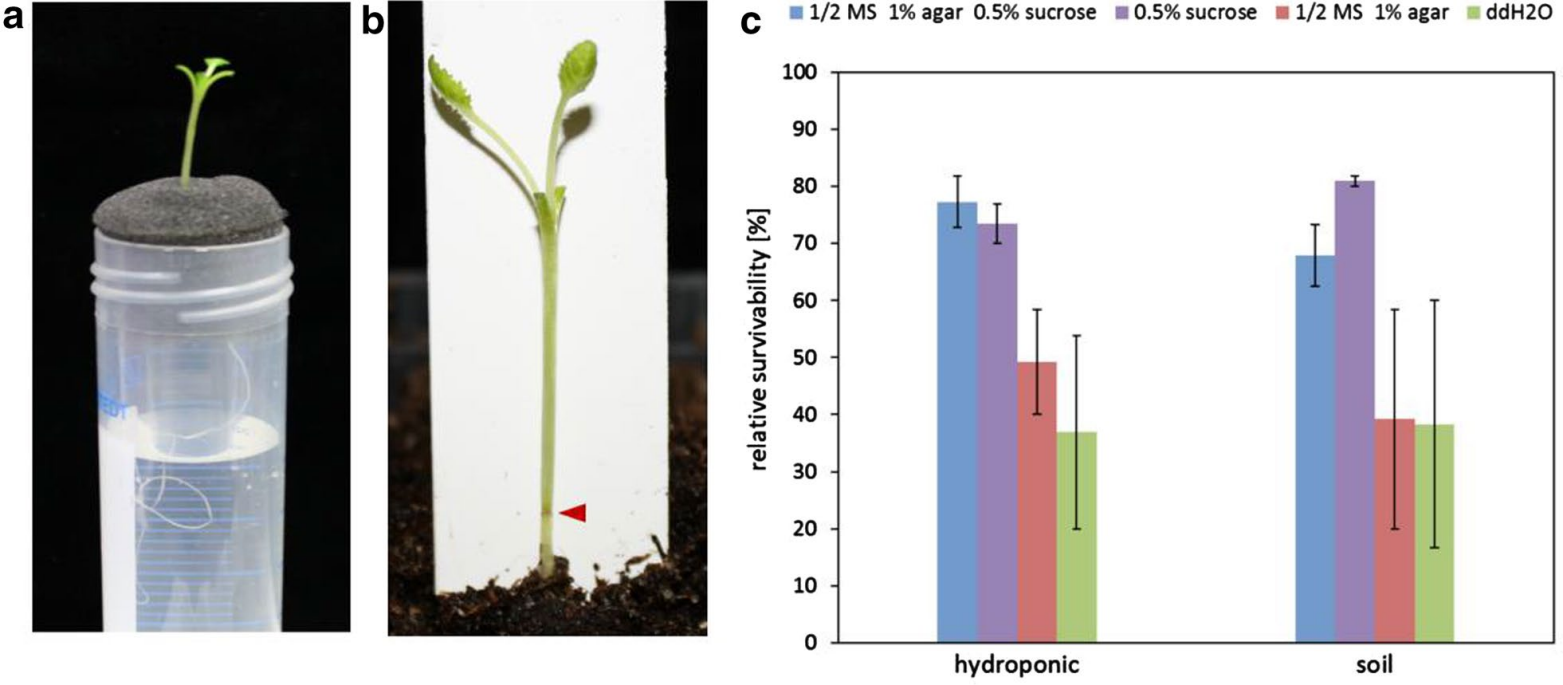
Post-grafting cultivation of 14 day old *B. napus* cv. Drakkar grafts. For further cultivation of grafts the plants were transferred to hydroponic culture or soil. For hydroponic cultivation the grafted plants were wrapped in black sponge, in which the graft junction was positioned in the middle and transferred to a 50 ml-Falcon tube consisting 40 ml medium (**a**). For cultivation on soil grafts were transferred to well-watered soil, in which the graft junction was free of soil (red arrow) and stabilized by a plastic plant label (**b**). Comparison of graft survival rates after hydroponic and soil cultivation (**c**). Survivability was determined after 14 days post-cultivation. ½ MS 1 % agar 0.5 % sucrose (hydroponic n = 22; soil n = 23); ½ MS 1 % agar (hydroponic n = 17; soil n = 18); ddH_2_O (hydroponic n = 18; soil n = 21; 0.5 % sucrose (hydroponic n = 23; soil n = 21)

As shown in Fig. 2b, there were significant differences in successful graft formation between conditions with and without additional sucrose. Sucrose-treated grafts showed higher survival rates (80–100 %) and a higher reproducibility when compared to sucrose-free conditions. Furthermore, the sucrose-treated grafts were much larger on agar plates and on filter paper, looked healthier, and the more stable graft connections allowed easier handling of grafts (Fig. 2a). Because of this, grafts treated with sucrose were more often successfully transferred to hydroponic culture or soil (Fig. 3a, b). Success rates ranged between 70 and 80 % (Figs. 2b, 3c). Again, regeneration without sucrose led to a high variability in survival rates after the transfer to either hydroponics or soil (Fig. 3c). The reason was that sucrose-treated grafts were less prone to breakage of the graft unions during transfer. This observation corresponds to results recently reported for *A. thaliana* where also 0.5 % sucrose improved grafting success in cotyledon-less grafts [24]. Adventitious root formation, as described to be problematic in Arabidopsis and other species [2, 12, 39], was no problem in Brassica grafts, no matter whether they were supplied with sucrose or not. Orienting the plates slightly vertically (5°–10°) increased grafting success by reducing bending of hypocotyls and thus facilitating orientation of the grafting partners, as also observed in Arabidopsis [24, 39]. Experiments performed with another *B. napus* cultivar (cv. Licosmos) confirmed the results obtained in cv. Drakkar, but Licosmos seedlings were in general smaller and even less delicate to handle (see Additional file 1: Figure S1, Additional file 2: Figure S2, Additional file 3: Figure S3).

In contrast to other published grafting procedures for *B. napus* [34–36] this work provides an easy and robust protocol for routine grafting with high success rates. Collar-free grafting is easier to handle and less laborious. When germination, grafting and graft generation are carried out under at least semi-sterile conditions, grafting success was significantly increased by minimising contamination of the graft junctions. Another advantage of our protocol is its applicability to mid- to high-throughput, as transport studies often require high numbers of grafted plants to allow statistically relevant conclusions.

In parallel experiments performed with Arabidopsis seedlings (data not shown) it could be observed that grafting on filter paper was more successful than grafting on agar plates. Also here, sucrose enhanced size and fitness of the grafts, but led to a slightly higher formation of adventitious roots in this species. Generally, too much moisture hindered successful formation of graft unions and water films on both agar plates and filter paper in all types of grafting experiments performed. A similar observation was made in recent Arabidopsis grafting experiments [24].

**Fig. 4:**
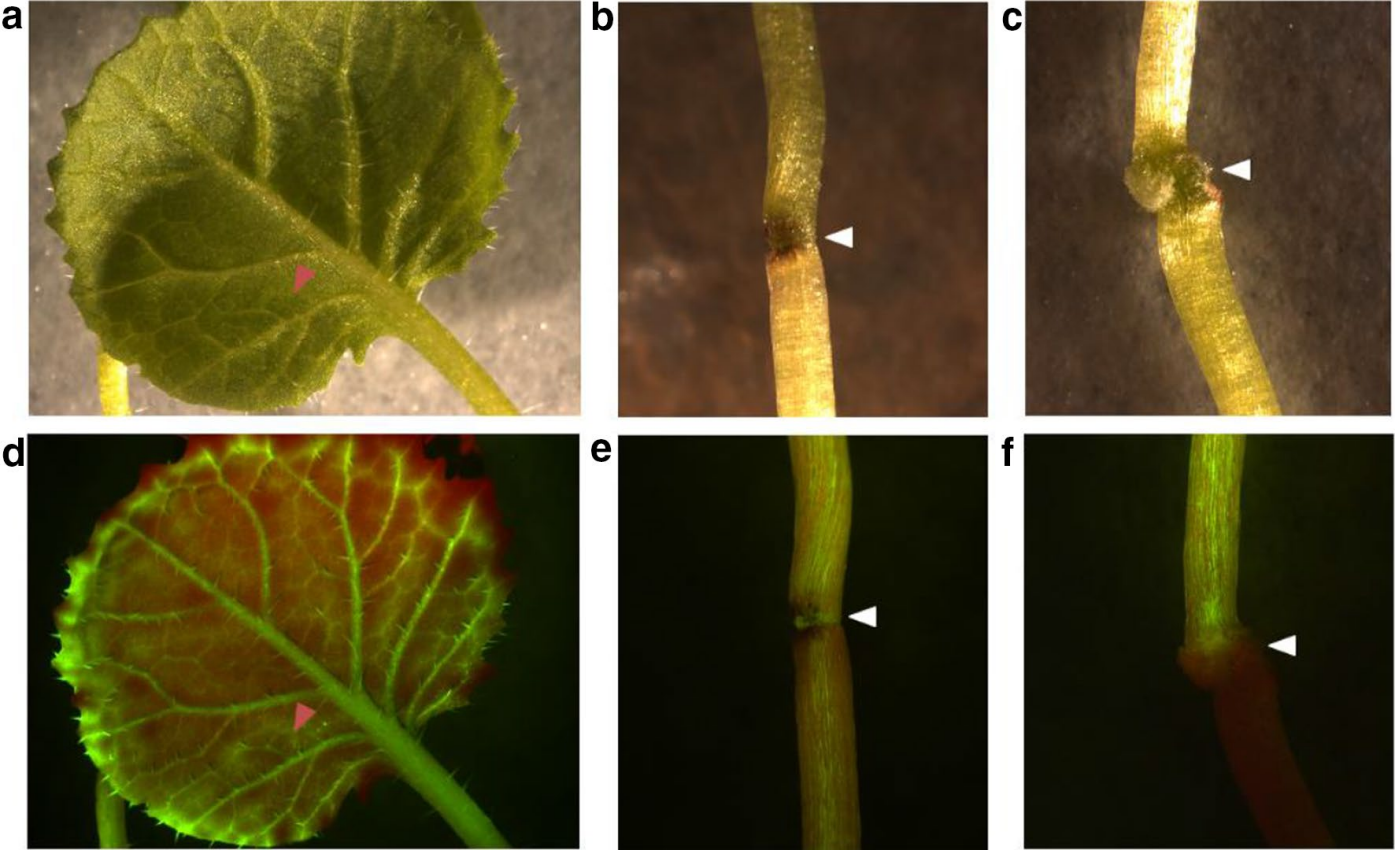
Analysing graft junction formation by CFDA staining. 14 day old grafts were transferred to a plate containing ½ MS and 1 % agar and stained with the phloem-mobile fluorochrome CFDA. Therefore a leaf of the grafts was punctured with a sterile needle (*red arrow*) and a few microliters of CFDA stain were applied at the point of lesion (**a**). After an incubation of 30 min at room temperature the plants were investigated under a fluorescence binocular using bright field (**a**–**c**) and fluorescence illumination with a GFP filter (**d**–**f**). While in successfully grafted plants the CFDA stain passed the graft junction (**e**), it accumulated in the scion part of the graft, when graft formation was not effective (**f**)

**Fig. 5:**
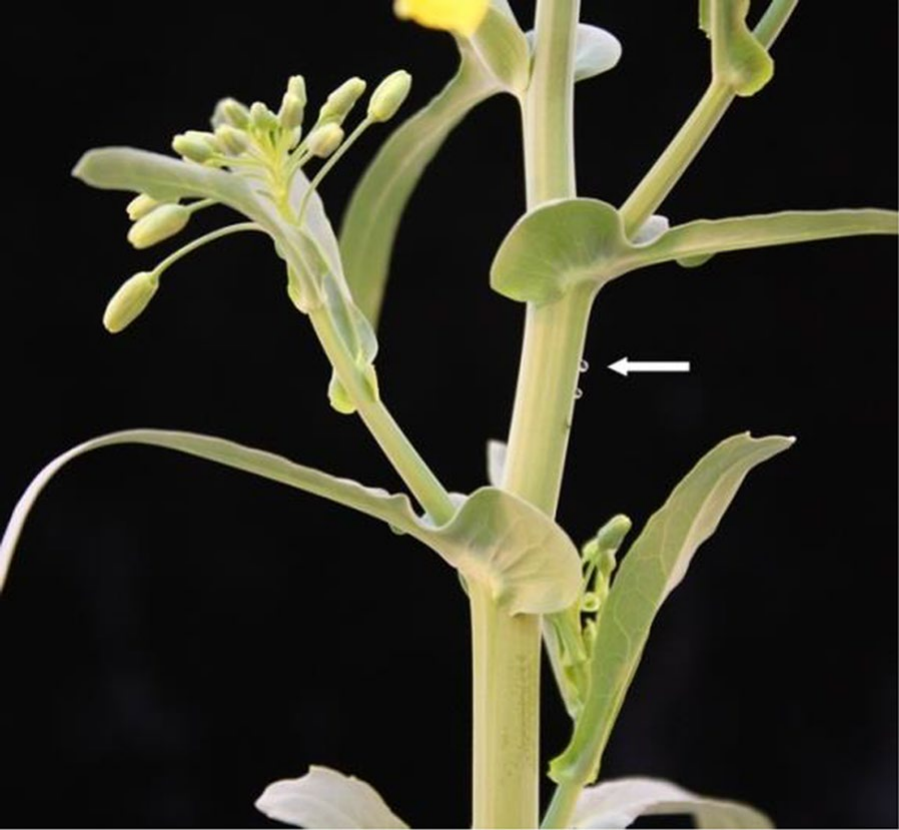
Phloem sap sampling from grafted *B. napus* plants. 10-week-old grafted plants were punctured with a hypodermic needle below the inflorescence as first described by Giavalisco et al. [28]. The *white arrow* indicates droplets of phloem sap after puncturing. For phloem sample collection the first droplet is discarded due to the danger of contamination by the content of injured cells. Several hundred microliters of phloem sap could be collected per plant independent on whether they were grafts or not

The establishment of functional vascular connections was first checked using the phloem-accumulating fluorescence dye CFDA. Figure 4 shows that most viable grafts disposed of functional vascular connections that allowed movement of CFDA, while a few did not allow long-distance movement of CFDA. These grafts did not survive long-term. Since one major aim of our study was to produce grafts for xylem and phloem sampling, we tested whether 10-week-old grafted plants allow sampling of xylem and phloem sap. An example of phloem sap exuding from a grafted plant is shown in Fig. 5. Exudation can be observed after small incisions were applied with a syringe needle as first described in Giavalisco et al. [28]. We could not detect any difference in xylem or phloem sampling (concerning e.g. sap quantity or duration of exudation) when comparing grafted with non-grafted plants, what demonstrates the suitability of our grafting protocol for long-distance transport studies.

## Conclusions

Grafting is a versatile tool to study long-distance mobility of potential signalling compounds. The development of optimised protocols that allow reliable grafting with high success rates is essential to obtain reasonable and reproducible results. In this study we present a simple and efficient grafting procedure for *B. napus* that in routine application allows a short-term survival rate of 80–100 % and still 70–80 % after transfer to hydroponic culture or soil, respectively. This demonstrates that *B. napus* is highly suitable for performing transport studies using the easy grafting procedure presented here. That is important, because *B. napus* is already used as a suitable model plant for xylem sap and phloem sap analyses due to the relatively easy access to quite large sample volumes from both long-distance transport systems. Long-term survival rates on hydroponic culture or soil enable the growth of grafts until the time-point suitable for phloem sap sampling. Therefore, in contrast to grafting studies in model plants like Arabidopsis, Brassica grafting studies do not only allow indirect conclusions of phloem mobility of potential signalling compounds, but their direct detection in collected phloem samples.

## Additional files

10.1186/s13007-016-0122-x Post-grafting cultivation of 14 day old *B. napus* cv. Licosmos grafts. Comparison of *B. napus* cv. Licosmos graft survival rates after hydroponic and soil cultivation. Survivability was determined after 14 days post-cultivation. ½ MS 1 % agar 0.5 % sucrose (hydroponic n = 40; soil n = 18); ½ MS 1 % agar (hydroponic n = 10; soil n = 10); ddH_2_O (hydroponic n = 28; soil n = 12; 0.5 % sucrose (hydroponic n = 10; soil n = 12).
10.1186/s13007-016-0122-x Development of *B. napus* cv. Licosmos after 20 days in hydroponic culture, regenerated under different conditions.
10.1186/s13007-016-0122-x Hydroponic post-grafting cultivation of grafted *B. napus* seedlings. Plants were wrapped with foam and up to 30 could be cultivated in 50-ml conical tubes in one polystyrene boxes covered with light-permissive plastic cover to prevent desiccation.
