# Supplementary material for: Protocol: optimisation of a grafting protocol for oilseed rape (Brassica napus) for studying long-distance signalling
Source: Plant Methods. 2016 Mar 25;12:22. doi: 10.1186/s13007-016-0122-x (PMC4807576; doi:10.1186/s13007-016-0122-x)
Supplement: Supplementary file 1 — 10.1186/s13007-016-0122-x Post-grafting cultivation of 14 day old B. napus cv. Licosmos grafts. Comparison of B. napus cv. Licosmos graft survival rates after hydroponic and soil cultivation. Survivability was determined after 14 days post-cultivation. ½ MS 1 % agar 0.5 % sucrose (hydroponic n = 40; soil n = 18); ½ MS 1 % agar (hydroponic n = 10; soil n = 10); ddH2O (hydroponic n = 28; soil n = 12; 0.5 % sucrose (hydroponic n = 10; soil n = 12). [file 13007_2016_122_MOESM1_ESM.docx]

**Additional file 1**

**
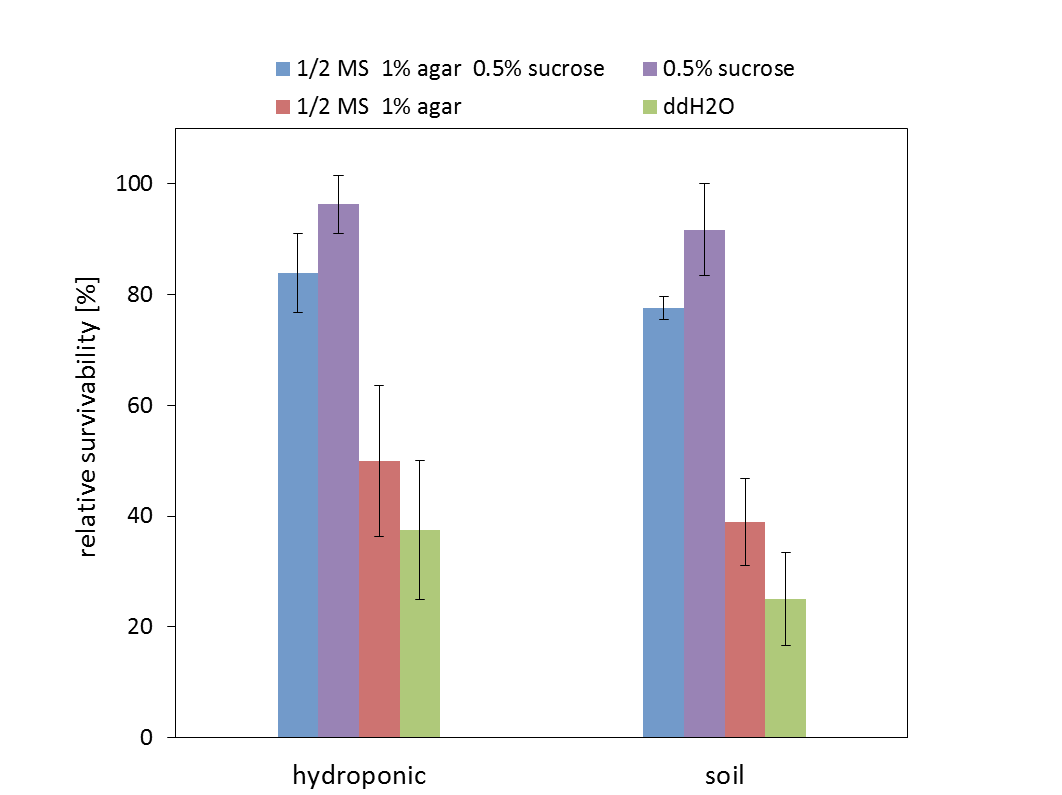
**

**Figure S1:** **Post-grafting cultivation of 14 day old *B. napus* cv. Licosmos grafts.** Comparison of *B. napus* cv. Licosmos graft survival rates after hydroponic and soil cultivation. Survivability was determined after 14 days post-cultivation. ½ MS 1% agar 0.5% sucrose (hydroponic n=40; soil n=18); ½ MS 1% agar (hydroponic n=10; soil n=10); ddH_2_O (hydroponic n=28; soil n=12; 0.5% sucrose (hydroponic n=10; soil n=12).
