# Supplementary material for: Protocol: optimisation of a grafting protocol for oilseed rape (Brassica napus) for studying long-distance signalling
Source: Plant Methods. 2016 Mar 25;12:22. doi: 10.1186/s13007-016-0122-x (PMC4807576; doi:10.1186/s13007-016-0122-x)
Supplement: Supplementary file 2 — 10.1186/s13007-016-0122-x Development of B. napus cv. Licosmos after 20 days in hydroponic culture, regenerated under different conditions. [file 13007_2016_122_MOESM2_ESM.docx]

**Additional file 2**

**
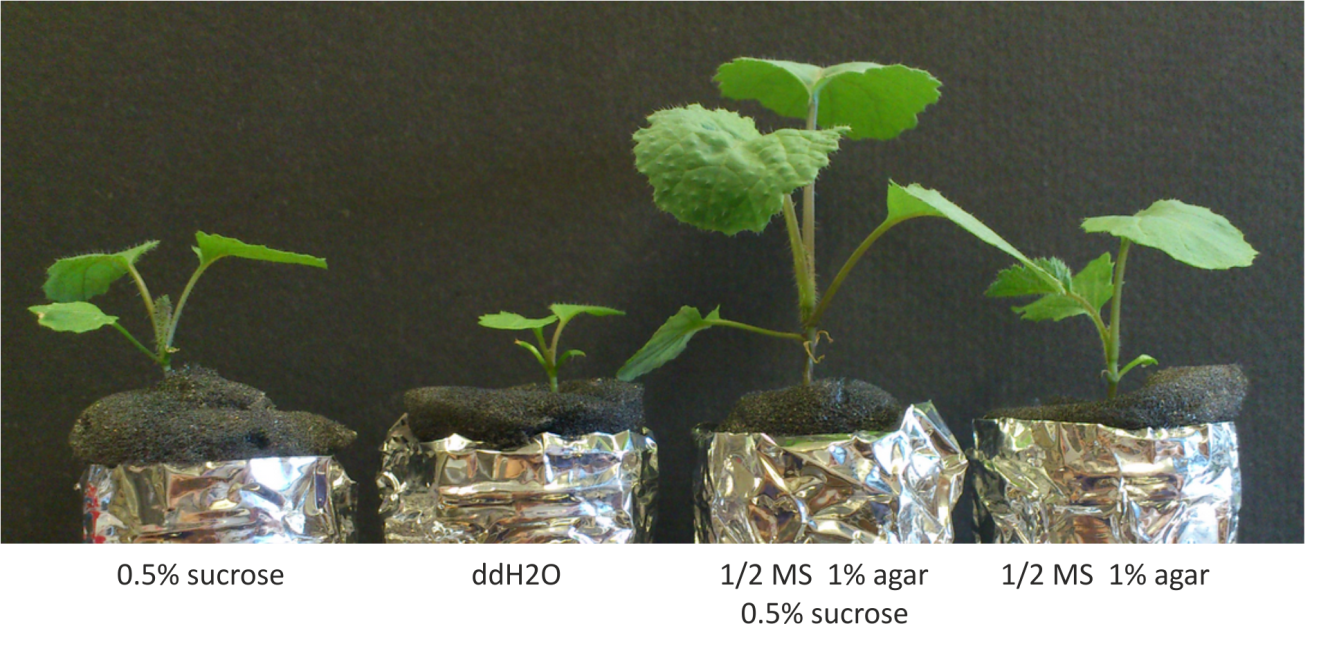
**

**Figure S2:** Development of *B. napus* cv. Licosmos after 20 days in hydroponic culture, regenerated under different conditions.
