# Supplementary material for: Protocol: optimisation of a grafting protocol for oilseed rape (Brassica napus) for studying long-distance signalling
Source: Plant Methods. 2016 Mar 25;12:22. doi: 10.1186/s13007-016-0122-x (PMC4807576; doi:10.1186/s13007-016-0122-x)
Supplement: Supplementary file 3 — 10.1186/s13007-016-0122-x Hydroponic post-grafting cultivation of grafted B. napus seedlings. Plants were wrapped with foam and up to 30 could be cultivated in 50-ml conical tubes in one polystyrene boxes covered with light-permissive plastic cover to prevent desiccation. [file 13007_2016_122_MOESM3_ESM.docx]

**Additional figure 3**

**
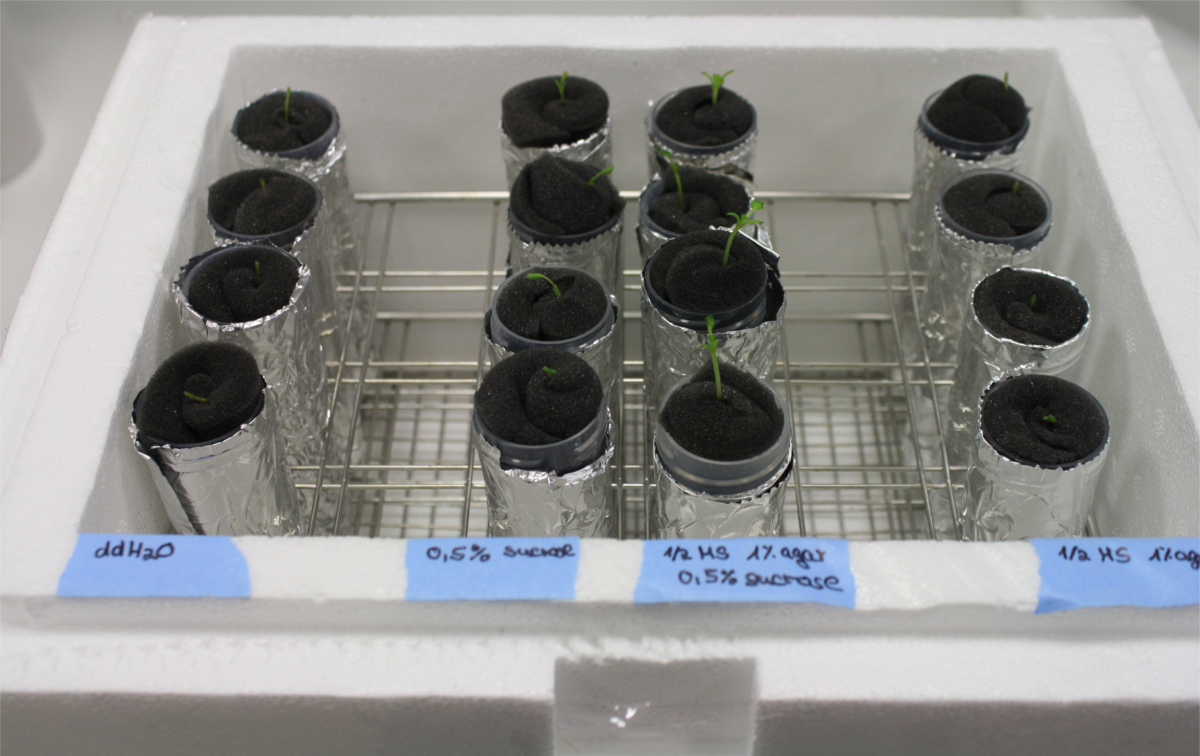
**

**Figure S3:** Hydroponic post-grafting cultivation of grafted *B. napus* seedlings. Plants were wrapped with foam and up to 30 could be cultivated in 50-ml conical tubes in one polystyrene boxes covered with light-permissive plastic cover to prevent desiccation.
